# Value of digital telethermography for the diagnosis of septic knee prosthesis: a prospective cohort study

**DOI:** 10.1186/1471-2474-14-7

**Published:** 2013-01-04

**Authors:** Carlo Luca Romanò, Roberto D’Anchise, Marco Calamita, Giovanni Manzi, Delia Romanò, Valerio Sansone

**Affiliations:** 1 Dipartimento di Chirurgia Ricostruttiva e delle Infezioni Osteo-articolari, Istituto Ortopedico I.R.C.C.S, Galeazzi - Via Riccardo Galeazzi, 4 - Milano, 20166, Italy; 2 Dipartimento di Chirurgia del Ginocchio, Istituto Ortopedico I.R.C.C.S, Galeazzi - Via Riccardo Galeazzi, 4 - Milano, 20166, Italy; 3 Istituto Ortopedico I.R.C.C.S, Clinica Ortopedica, Galeazzi - Via Riccardo Galeazzi, 4 - Milano, 20166, Italy

**Keywords:** Knee, Total knee replacement, TKR, Infection, Thermography, Diagnosis

## Abstract

**Background:**

Diagnosis of peri-prosthetic infection remains challenging, often requiring a combination of different tests.

**Methods:**

In this prospective, case–control study, the diagnostic accuracy of telethermography was evaluated in a group of seventy patients who had had a total knee replacement and were undergoing a reoperation because of infection or another implant-related problem, after a minimum of one year from implant.

**Results:**

An average differential temperature of the affected versus not affected knee of 1.9°C was observed in infected prosthesis, compared to 0.3°C in aseptic failures. Considering a normal reference value equal or less than 1.0°C, telethermography showed an accuracy, sensitivity, specificity, positive and negative predictive value of, respectively: 0.90, 0.89, 0.91, 0.91, 0.88.

**Conclusions:**

Digital telethermography is a reliable option for diagnosing peri-prosthetic knee infection.

## Background

Infection has been recently reported as the first reason for revision after total knee replacement in the U.S.A. [1], however, since no single test has been proved to be 100% sensitive and specific, the diagnosis of septic failure remains frequently challenging and a combination of multiple tests is often required to rule out or to confirm the suspect of peri-prosthetic infection in a painful knee prosthesis [2-4].

Infra-red thermography has been shown to detect temperature changes associated with many different diseases [5-10] and for post-operative monitoring of surgical site healing in various clinical settings [11-15].

After the first report from Lambiris and co-workers, three decades ago, that described the early thermographic changes at the surgical site following orthopaedic surgery [16], only few others authors investigated the use of this technology for the diagnosis of bone and joint infections [17-19].

Infra-red digital telethermography (IRDT) may now be performed through newly available, digital telethermocameras [20], that do offer portability, ease of use even for the non specialized personnel, precise and real-time measurements, at relatively low costs. Using this technology, we recently investigated the physiological telethermographic pattern of wound healing after total knee and hip prosthesis and a short series of patients affected by peri-prosthetic late infection after total knee replacement [21,22].

Purpose of this prospective study was to assess the ability of IRDT to differentiate septic versus aseptic painful total knee arthroplasties (TKA).

## Methods

During years 2009–2010, 70 patients with painful TKA and scheduled for total knee revision surgery, were included in this prospective, observational, case–control study.

Exclusion criteria were: time from knee prosthesis implant less than one year, any knee surgery or trauma after the index operation, rheumatological disorders.

Reasons for TKA implant were: primary osteoarthritis (51 patients), post-traumatic osteoarthritis (12), osteonecrosis (7).

The present research has been carried out in compliance with the Helsinki Declaration on human rights. Local IRB (Direzione Scientifica Istituto Ortopedico Galeazzi) approval was obtained prior to the start of the study, partially funded by the Italian Ministry of Health (research project no. 4065/09). All the patients gave their written informed consent to participate in the study and to the publication of clinical images.

### Patients assessment

Pre-operative clinical examination to investigate local signs of inflammation (swelling, redness, warmth, stiffness, draining sinuses) was undertaken in all the patients. Pain at rest and at movement (knee flexion and extension) was recorded using the visual analogue scale (V.A.S.), asking the patient to rate his/her pain on a 10 cm scale (0 no pain – 10 maximum tolerable pain).

Laboratory tests include serum levels of C-reactive protein (C-RP), erythrocyte sedimentation rate (ESR), interleukin-6 (IL-6) and the white blood-cell count (WBC); routine radiographic evaluation had also been performed in all the patients.

Positive diagnosis of peri-prosthetic infection was made according to the presence of one or more of the following parameter:

1) Draining sinus;

2) Positive pre-operative joint aspiration cultures;

3) Positive intra-operative cultures (at least two of five samples). Sonication on the retrieved material was used in all the cases;

4) Positive histological finding (> 5 leucocyte per field).

### Telethermographic data acquisition and processing

Infra-red thermal images were acquired using the NEC-AVIO ThermoShot F30S digital telethermocamera. Specifications of the camera are the followings: measurement range: -20 to 100°C; temperature resolution: 0.1°C (at 30°C), better than 0.1°C with averaging; wavelength: 8 – 13 μm; spatial resolution: 3.1 mrad; measurement distance: 10 cm to infinite dimensions: 100 × 65 × 45 mm; weight: 350 g, including rechargeable batteries.

Thermographic images were taken with the patient laying supine, the two legs slightly apart, on the day before surgery (first session of data acquisition) and on the same day of surgery (second session); one of two different investigators was randomly assigned to the first or to the second session data acquisition to assess inter-observer variability.

Both the knees were included in the same shot and the thermographic values of the two knees were compared. Five shots were taken during each session, discarding the first one, while using the remaining four for further analysis. Only the anterior aspect of the knees was investigated. No efforts were made to keep the ambient temperature or humidity at constant level, as all those parameters are automatically recorded by the digital camera and were considered to equally affect both limbs at the time of temperature recording. Instead, care was taken as to leave the knees uncovered for at least three minutes before each recording and to avoid liquid dressings and direct spot-lights on the joint during image acquisition.

### Telethermographic data processing

**Figure 1:**
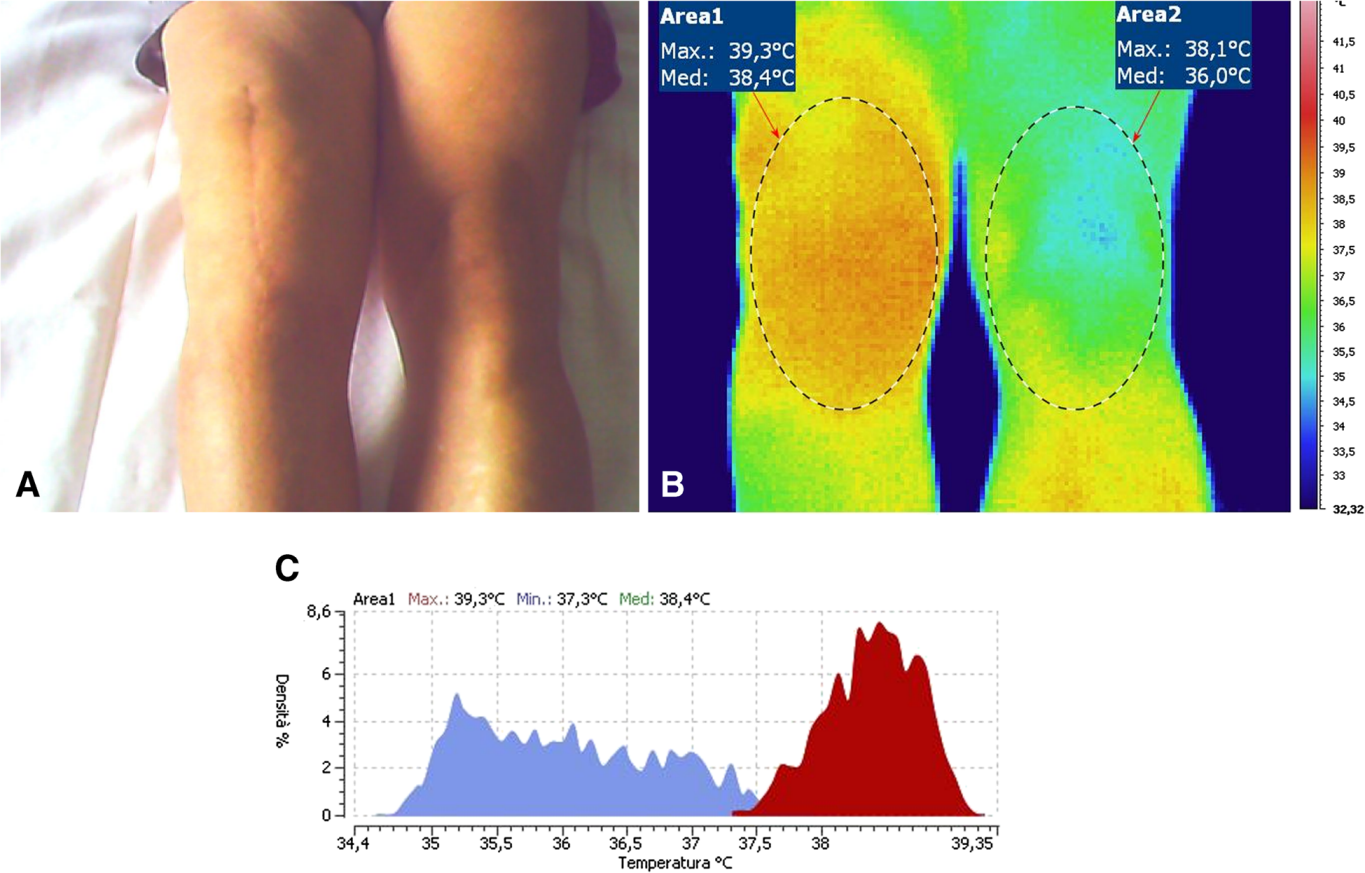
**Right knee infected prosthesis.** Clinical aspect (**A**) and thermogram (**B**), with maximum (Max) and average (Med) recorded temperature and temperature distribution (**C**).

**Figure 2:**
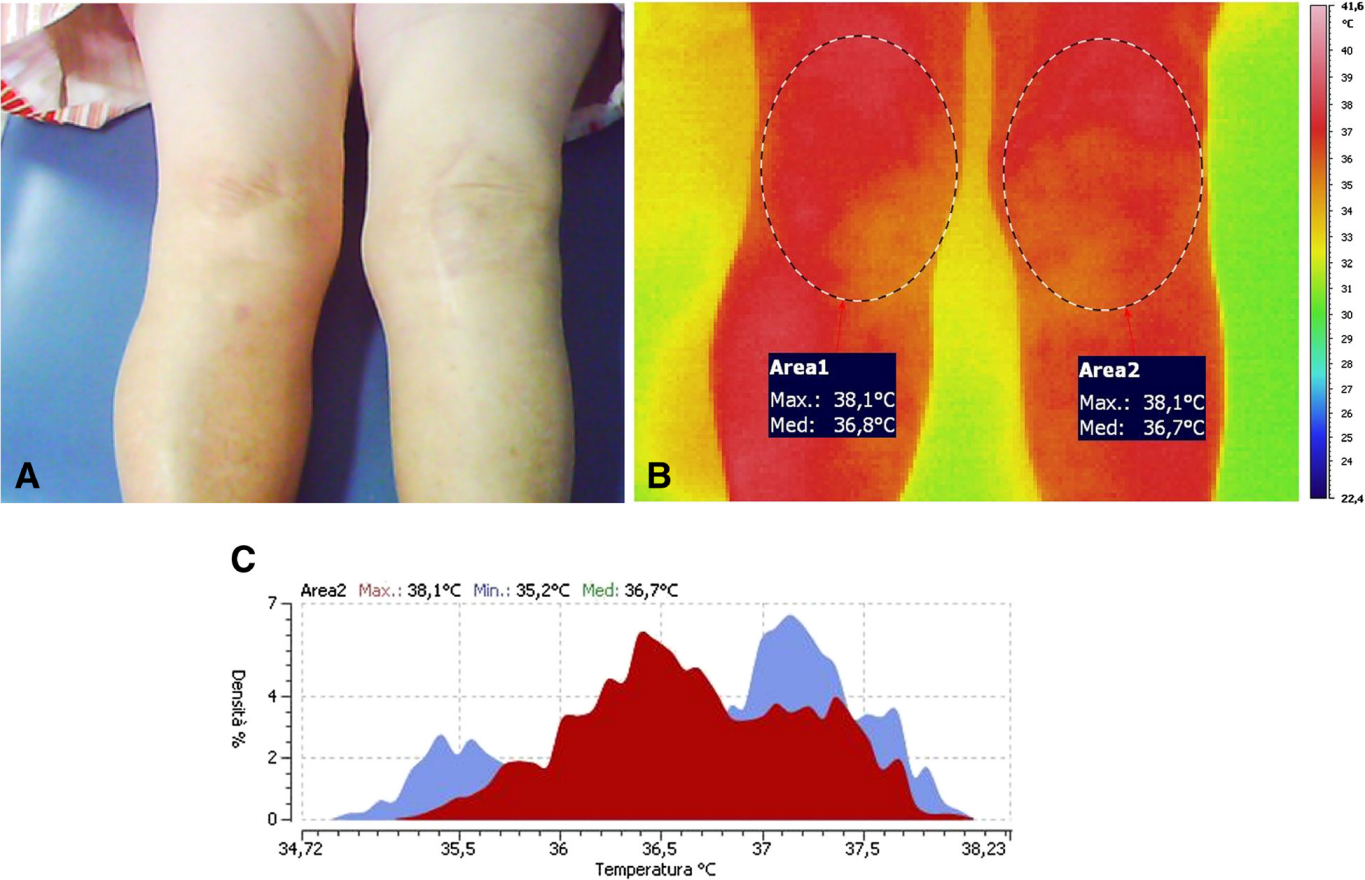
**Aseptic failed left knee prosthesis.** Clinical aspect (**A**) and thermogram (**B**), with maximum (Max) and average (Med) recorded temperature and temperature distribution (**C**).

Data processing had been conducted with the dedicated software IRTCronista by an investigator not aware of the results of the clinical and laboratory tests. The software allows drawing an elliptical area (Surgical Site Area, SSA) on the anterior aspect of each knee (Figures 1 and 2), with the major axis measuring approximately 20 cm on the midline of the knee and the minor axis of approximately 12 cm crossing the major axis in its center. The peak temperature within the SSA (Hottest Spot, HS) and the average temperature of any given SSA can be calculated automatically by the same software. For further analysis, we then considered the differential temperature (affected minus not-affected knee) of the HS and of the SSA.

Data from each acquisition session were used to assess intra-observer variability, while the average data obtained during the first and the second sessions were compared to assess inter-observer variability.

Data from the first acquisition session were then used for sensitivity, specificity and predictive value assessment.

### Statistical analysis

Statistical analysis was performed using the unpaired *t* test for continuous series of data (http://www.graphpad.com/quickcalcs/ttest1.cfm).

## Results

Of the 70 patients scheduled for knee revision surgery, 36 were diagnosed as being peri-prosthetic infections and 34 as aseptic implant failures (9 were aseptic loosening due to component wear, 9 prosthesis malpositioning, 6 anterior knee pain, 7 persistent pain of unknown origin, 3 stiff knees). Mean age at admission to our hospital was, respectively, 66.8 years (range, 54–77 years) and 68.9 years (range, 53–80 years) in the septic and aseptic groups. Time from implant ranged from 1 to 10 years (mean 2.1 ± 1.6 years) in the infected prosthesis group, compared to 1 to 12 years (mean 3.6 ± 2.1 years) in the aseptic failed knees.

Causative pathogens were identified in 28 patients (77.8%), in 6 cases only prior to surgery through joint aspiration, in 16 only intra-operatively and in 6 patients both pre- and intra-operatively. Gram positive cocci were isolated in approximately 70% of cases (cf. Table 1).

**Table 1:** Isolated pathogens the septic group of patients (N = 36)

	**Raw numbers**	**%**
CNS	10	27.8
MRSE	3	8.3
MSSA	8	22.2
MRSA	5	13.9
Pseudomonas aeruginosa	4	11.1
Enterococcus spp.	3	8.3
E. coli	1	2.7
Pasteurella multocida	1	2.7
No isolates	8	22.2

CNS: coagulase-negative staphylococci; MRSE: Methicillin-Resistant Staph. Epidermidis; MSSA: Methicillin-Sensitive Staph. Aureus; MRSA: Methicillin-Resistant Staph. Aureus (more than one microrganism may be isolated form a given patient).

Mean pain at rest in the septic and aseptic failed prosthesis was, respectively, 23.6 ± 11.9 (range 0 – 52) and 23.5 ± 14.5 (0 to 45), while all the patients complained about pain at weight bearing and at movement, ranging from 15 to 80 (mean: 52.2 ± 14.6) in the septic group and from 25 to 74 (44.2 ± 17.3) in the aseptic failed prosthesis. Other clinical signs of infection were inconstant (cf. Table 2).

**Table 2:** Pre-operative clinical assessment of the reviewed patients (N = 70) (more than one sign may be present in a patient)

	**Infected TKA (N = 36)**	**Aseptic failed TKA (N = 34)**
Swelling	26 (72%)	18 (53%)
Local redness	22 (61%)	5 (15%)
Local warmth (clinical perception)	22 (61%)	7 (20%)
Stiffness	10 (28%)	11 (32%)
Draining fistula	7 (19%)	0
Fever	3 (8%)	0

Mean values of C-RP, ESR and IL-6 were significantly different among septic and aseptic failed prosthesis, as reported in Table 3. Osteolysis and/or radiolucent lines around the prosthesis was a common, although not specific, finding (19/36 in infected prosthesis versus 24/34 in aseptic failures).

**Table 3:** Pre-operative laboratory tests (N = 70)

	**Infected TKA (N = 36)**		**Aseptic failed TKA (N = 34)**		**P**
	**Mean ± S.D.**	**Range**	**Mean ± S.D.**	**Range**	
C-RP (mg/L)	38.4 ± 41.2	2 - 135	6.4 ± 7.1	0 – 22	0.0001
ESR (mm/hr)	52 ± 43	15 - 160	11 ± 18	4 – 46	0.0001
WBC / ml	7400 ± 2200	4200 - 13700	6500 ± 2200	4200 – 13700	0.09
IL-6 (pg/ml)	8.8 ± 3.8	4.9 – 12.2	2.9 ± 0.4	2.8 - 3.2	0.0001

Figures 1 and 2 show the clinical aspect and the typical thermograms of, respectively, a septic and an aseptic failed knee prosthesis. The temperature distribution in the affected and the sound knee showed two distinct curves and peaks in infected implants (Figure 2C) while the two curves usually overlaps in the aseptic failed prosthesis, (Figure 2C).

**Figure 3:**
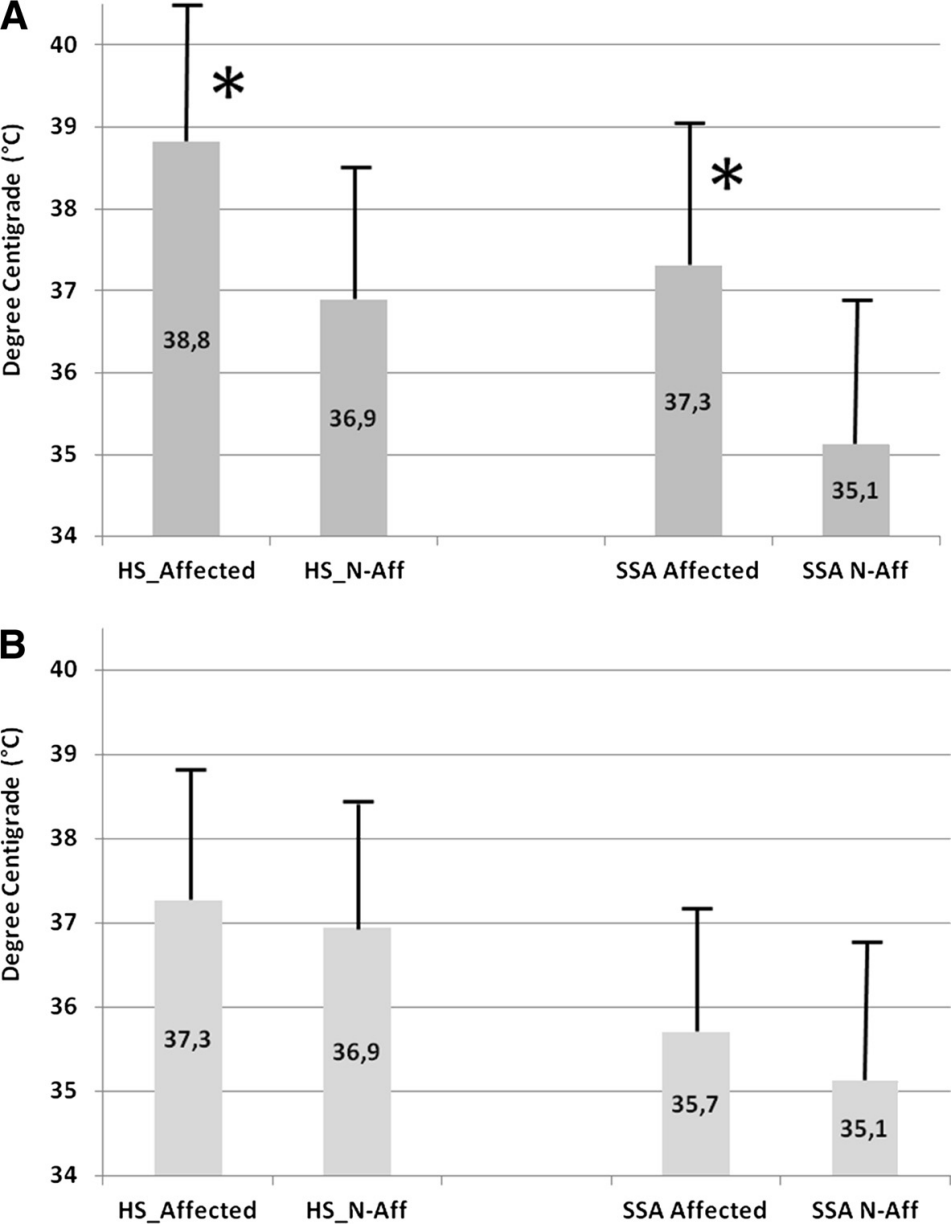
**Mean recorded temperatures of septic (A) and aseptic (B) failed knee prosthesis.** HS: Hottest Sport. SSA: Surgical Site Area. Affected: painful knee. N-Aff: sound knee. * P < 0.0001.

Mean recorded Hottest Spots (HS) and Surgical Site Areas (SSA) are reported in Figure 3. In infected prosthesis (Figure 3A), both the HS and the SSA mean recorded temperatures were statistically higher in the affected knee, compared to the sound joint, while in aseptic failed implants (Figure 3B) the difference was not statistically significant.

The mean differential HS temperature (affected minus not affected knee values) were, respectively, 1.92 ± 1.20°C and 0.32 ± 0.73°C (P < 0.0001) in the septic and aseptic knee prosthesis; similarly, mean differential SSA temperatures were, respectively 2.17 ± 1.47°C and 0.58 ± 0.48°C (P < 0.0001).

**Figure 4:**
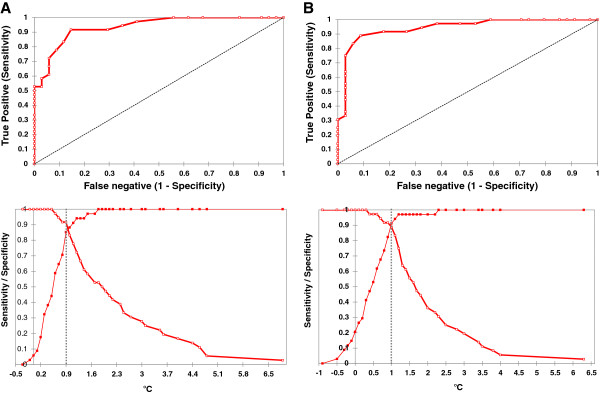
**Receiver Operating Curve (ROC) for temperature threshold assessment for Surgical Site Area (A) and Hottest Spot (B).** Dotted lines indicate the temperature at which the highest value of sensitivity + specificity was calculated.

Receiver Operating Curves (ROC) were calculated to assess the best temperature threshold for differential diagnosis, both for the Surgical Site Area (Figure 4A, area under the curve: 0.937 + − 0.021; 95% C.I.: 0.89 – 0.98) and for the Hottest Spot (Figure 4B, area under the curve: 0.942 + − 0.023; 95% C.I.: 0.90 – 0.99); according to the calculations, a value equal or less than 0.9°C provided the highest sensitivity + specificity value (1,770) and precision (0,886) for SSA, while a value equal or less than 1.0°C provided the highest sensitivity + specificity value (1,801) and precision (0,900) for the HS.

Considering as normal reference value (no infection) a differential Hottest Spot temperature between the affected and not affected knee equal or less than 1.0°C, we observed three false positive and four false negative results with an accuracy of 0.90 and a sensitivity, specificity, positive and negative predictive value of, respectively: 0.89 (95% C.I.: 0.74 – 0.96), 0.91 (95% C.I.: 0.76 – 0.98), 0.91 (0.78 – 0.97) and 0.88 (0.74 – 0.95).

The differential SSA temperature provided the best accuracy (0.88) with a reference normal value equal or less than 0.9°C, with a sensitivity, specificity, positive and negative predictive value of, respectively: 0.92 (95% C.I.: 0.77 – 0.98), 0.85 (95% C.I.: 0.69 – 0.94), 0.87 (0.72 – 0.94) and 0.91 (0.76 – 0.97).

Intra - and inter -observer variability in measuring the differential HS temperatures were, respectively, on average 0.2 ± 0.3°C and 0.3 ± 0.3°C.

## Discussion

Differential diagnosis between septic and aseptic painful knee prosthesis may be challenging. This is the first study reporting on the diagnostic value of telethermography to diagnose peri-prosthetic knee late infections and to differentiate these from aseptic failures. We showed that, in this series of patients, aseptic failure of a knee prosthesis is not associated with a significant increase in local temperature, at variance to that one can observe in a peri-prosthetic infection. This study provides evidence that thermography, using a digital, portable, telethermocamera and a dedicated software, is a rather accurate, reproducible and reliable test to differentiate septic and aseptic painful knee prosthesis. Our data also point out that the information provided by the analysis of the Hottest Spot is not inferior to that of the average temperature of the Surgical Site Area.

However, the following limitations of this study should be taken into consideration:

– The value of telethermography has only been investigated at a minimum of one year after surgery. Previous analysis showed a reproducible pattern in the course of temperature at the surgical site, with a peak of temperature the first days after surgery [14-19] and we have previously reported the “physiological” telethermographic pattern of surgical site healing after total hip and knee replacements and the long time needed to return to baseline values [21,22]. For this reason we decided only to include patients with a painful knee prosthesis after a minimum of one year after implant;

– We only investigated the anterior aspect of the knee. Maybe further information could be obtained through side and posterior thermal acquisitions;

– Intra- and inter-observer variability represent a possible source of diagnostic error and may limit the reliability of this technology, especially in a large scale use. To reduce this possible source of error, some training before the use of the telethermographic camera appears mandatory. Since fluctuations of the recorded temperatures are common even in the same recording session, multiple temperature acquisitions according to a fixed protocol seems also necessary;

– The criteria used in this study as a reference to define a peri-prosthetic infection were, to some extent, arbitrarily chosen, since no golden standard or universally accepted definition of peri-prosthetic joint infection exists at the moment; however, the criteria used in the present study, appear to be in line with those reported in the most recent literature [23].

– The series of patients is relatively small.

## Conclusions

This study shows that advantages of telethermovision in observing temperature in painful TKAs are manifold. The method is reproducible, painless, safe, non-invasive and gives an absolutely accurate image of temperature over a given area, while the comparative analysis of the thermograms of the two joints, with dedicated software, is relatively simple.

Modern digital telethermocameras are lightweight, portable, easy to use even from non specialized personnel, relatively inexpensive and appear a good candidate as a large scale screening and monitoring tool in the hospital department as well as in the physician’s office for painful total knee prosthesis.

## Competing interests

None of the authors has any financial or non-financial competing interests in relation to this manuscript.

## Authors’ contributions

DR, GM and MC participated in the surgical and medical treatment, wrote the draft of the manuscript and participated in the follow-up examination of the patient and clinical material. RDA and VS performed the surgical and medical treatment and followed up the patient. They also have been involved in drafting the manuscript and revising it critically. CLR performed the surgeries, coordinated and helped to draft and finalize the manuscript. All authors read and approved the final manuscript.

## Authors’ information

CLR is the Director of the Centro di Chirurgia delle Infezioni Osteo-articolari of the research orthopaedic institute Galeazzi in Milano, Italy. Past–president of the Italian Studygroup on Osteoarticular Infecitons, he actually serves as President of the European Bone and Joint Infection Society.

## Pre-publication history

The pre-publication history for this paper can be accessed here:

http://www.biomedcentral.com/1471-2474/14/7/prepub
